# Prognostic and Predictive Significance of PD-L1 Expression in Non-Small Cell Lung Cancer Patients: A Single-Center Experience

**DOI:** 10.5146/tjpath.2021.01545

**Published:** 2021-09-15

**Authors:** Hacı Arak, Aydın Aytekin, Ozlem Canoz, Metin Ozkan

**Affiliations:** Department of Medical Oncology, Gaziantep University School of Medicine, Gaziantep, Turkey; Department of Pathology, Erciyes University School of Medicine, Kayseri, Turkey; Department of Medical Oncology, Erciyes University School of Medicine, Kayseri, Turkey

**Keywords:** Lung, Non-small cell cancer, PD-L1, H-score.

## Abstract

***Objective:*** To investigate the prognostic and predictive value of PD-L1 expression in operated non-small cell lung cancer (NSCLC) patients and to analyze its relationship with clinicopathological factors.

***Material and Method:*** A total of 90 patients with operable NSCLC were included in this retrospective single center study. Tumor blocks of patients were stained immunohistochemically with PD-L1 polyclonal antibody. When evaluated immunohistochemically and statistically, patients with tumor staining percentage of ≥5%, those with +2 and +3 membranous staining intensity, and those with ≥50% H-Score were considered positive. The relationship between PD-L1 expression status and clinicopathological features in addition to the prognostic effect of PD-L1 on survival were statistically analyzed.

***Results:*** The frequency of PD-L1 expression was 37%, 15% and 5% according to the staining percentage, staining intensity, and the H-Score, respectively. There was no significant relationship between PD-L1 expression and age, gender, smoking, tumor stage and histological subtype (p> 0.05). However, PD-L1 expression was relatively higher in patients <65 years of age, men, smokers, patients with advanced tumor stage, and squamous cell subtype. Based on the analysis of the H-Score, no significant difference was noted regarding disease-free survival time between PD-L1 positive and PD-L1 negative patients (median 20 [95% CI 1.2-38.7] months vs. median 27 [95% CI 17.5-36] months, p=0.208). However, overall survival time was significantly shorter in PD-L1 positive compared to PD-L1 negative patients (median 24 months [95% CI 9.9-38] vs. median 48 months [95% CI 33.6-62.3], p=0.049).

***Conclusion:*** In patients with high PD-L1 expression, the biological behavior of the cancer was more aggressive, and the life expectancy was shorter. PD-L1 expression seems to be a poor prognostic marker in NSCLC patients.

## INTRODUCTION

Lung cancer, accounting for 18.4% of cancer-related deaths in the world, is the leading cause of cancer-related death in men, and the second most common cause of cancer-related death in women (1). Non-small cell lung cancer (NSCLC) constitutes 80% of all lung cancers, and most patients with NSCLC are diagnosed at an advanced stage, with a 5-year survival rate of 19% (2). Although the introduction of platinum-based chemotherapies and drugs for driver mutations has enabled a survival advantage in subgroups of patients, more effective and general treatments are needed given the continued progression of metastatic NSCLC disease (3).

In recent years, immune system modulation has been increasingly addressed in cancer treatment, particularly tumor cell escape from the attack of the immune system via inhibition of immune checkpoint molecules (4). The best known signaling pathways of immune checkpoints are T-lymphocyte antigen-4 and programmed cell death-1 (PD-1) / programmed cell death ligand-1 (PD-L1) (5). The co-inhibitory factor PD-1 binds to its ligands, PD-L1/PD-L2, to transmit inhibitory signals in T cells and anti-apoptotic signals in tumor cells. Thus, the PD-1/PD-L1 pathway is characterized as one of the major mechanisms of tumor immune escape. The PD-1/PD-L1 axis is also a physiological signaling pathway that causes depletion and inactivation of T cells to prevent the autoimmune response (6).

Immunotherapy with checkpoint inhibitors developed for the PD-1/PD-L1 pathway has become a new method in lung cancer treatment. Immunotherapy aims to ensure recognition of cancer cells by the immune system, increased sensitivity of the immune system, and reduction in immune system inhibition. Nevertheless, the same response cannot be obtained in all patients. PD-L1 expression level and clinicopathological characteristics of the patients may be a determining factor (7).

PD-L1 expression is a poor prognostic factor in cancers such as stomach cancer, hepatocellular carcinoma, renal cell carcinoma, esophageal cancer, pancreatic cancer, ovarian cancer, and bladder cancer (8). Conversely, PD-L1 expression is associated with better clinical outcomes in breast cancer and Merkel cell carcinoma. The prognostic value of PD-L1 expression in NSCLC, colorectal cancer, and melanoma is controversial (9). Additionally, the association of PD-L1 expression with clinicopathological factors in NSCLC patients is still unclear. Therefore, this study was designed to determine the prognostic and predictive value of PD-L1 expression in NSCLC patients, and to analyze the relationship between PD-L1 expression and clinicopathological factors.

## MATERIAL and METHODS

### Study Population

A total of 90 patients with NSCLC were included in this retrospective single center study, conducted at a tertiary care oncology clinic between May 2008 and September 2015. The diagnosis of an operable NSCLC, absence of a metastasis, and availability of sufficient tumor material for immunohistochemical analysis of PD-L1 expression were the inclusion criteria of the study.

The study was approved by the local Ethics Committee and was conducted in accordance with the ethical principles stated in the most recent version of the Declaration of Helsinki. (Institutional Ethics Committee Decision No: 2015/422).

### Evaluation of Clinicopathologic Characteristics

Baseline characteristics of patients (age, sex, smoking history, tumor stage, histologic type, and Eastern Cooperative Oncology Group [ECOG] performance status), treatment history (surgery, chemotherapy and radiotherapy), and clinical outcomes (survival, disease progression) were retrieved from hospital records. Pathologic tumor stage was defined based on the American Joint Committee on Cancer Cancer Staging Manual, 8th edition (10). The overall survival (OS) of the patients was calculated as the time between the date of diagnosis and last control or death due to disease. Disease-free survival (DFS) time was calculated as the time between the date of diagnosis and the date of relapse or last control or death.

### Immunohistochemical Procedure

For immunohistochemical staining, four-micrometer-thick histologic sections were cut from neutral buffered formalin-fixed, paraffin-embedded tissue blocks containing the sufficient tumor tissue. Before staining with PD-L1 antibody, various dilution ratios were tested to find the appropriate dilution ratio in a preparation and the appropriate dilution was determined as 1/200. Four micron-sections from each tumor block were taken on lysine glass. The sections were kept in the oven at 60 degrees. PD-L1 primary antibody (*CD274 Polyclonal antibody, Catalog no: EAP 0528, Rabbit, IgG, Abcam, Cambridge, United Kingdom*) was applied at 1/200 dilution. Staining was done automatically using the Ventana Benchmark XT immunohistochemistry stainer (*Roche, Mannheim, Germany*) (11). The stained slides were passed through series of alcohol with increasing ratios (70%, 90%, and 96%) for 5 minutes each. The stained slides were evaluated under the light microscope.

### Immunohistochemical Evaluation

**Figure 1:**
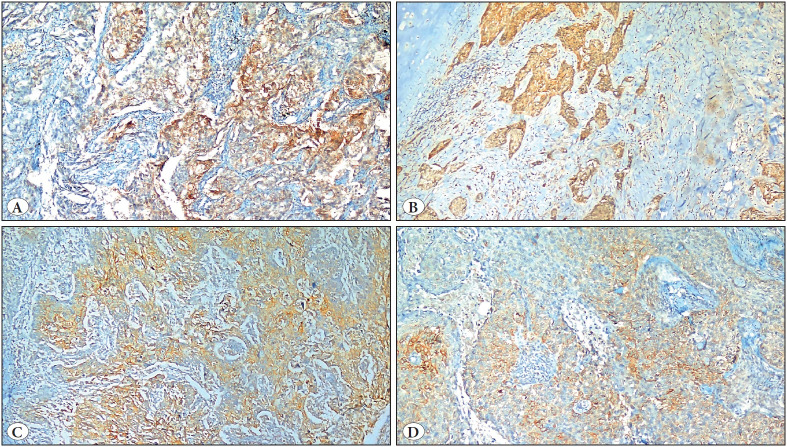
Percentage of staining and staining intensity with PD-L1 in NSCLC patients with various stages and histological subtypes. **A)** Tumor staining percentage of 25%, staining density + 3 histomorphological appearance of adenocarcinoma stage-1B case immunostained with PD-L1 (Anti-PDL1 antibody, X20). **B)** Tumor staining percentage of 30%, staining density + 2 histomorphological appearance of epidermoid cancer stage-2B case immunohistochemically stained with PD-L1 (Anti-PDL1 antibody, X10). **C)** Tumor staining percentage of 20%, staining density + 2 histomorphological appearance of an epidermoid cancer stage-3B case stained with PDL1 immunohistochemically (Anti-PDL1 antibody, X20). **D)** Tumor staining percentage of 10%, staining density + 3 histomorphological appearance of an epidermoid cancer stage-3A case immunohistochemically stained with PD-L1 (Anti-PDL1 antibody, X20).

The percentage of staining in the tumor was considered as ≤1% (negative), 1-5% (weak positive), 5-10% (medium positive), and 10-100% (strongly positive). For membranous staining intensity, the score was accepted as 0: negative, 1: weakly positive, 2: medium positive, and 3: strongly positive. According to the membranous staining intensity, PD-L1 0+ and 1+ were considered negative, 2+ and 3+ were considered positive. The H-Score was obtained via multiplying the tumor staining percentage and membranous staining intensity values and ranged from 0 to 300. While evaluating statistically, patients with a tumor staining percentage ≥ 5%, those with +2 and +3 membranous staining, and those with ≥50% H-Score were considered positive (12). The staining percentage and staining intensity of some patients are shown in Figure 1.

### Statistical Analyses

Statistical analysis was performed using IBM SPSS Statistics for Windows, version 21.0 (*IBM Corp., Armonk, NY*). Categorical variables were analyzed with the Chi-Square test and Fisher’s exact test, while Student’s t test was used to analyze parametric variables. DFS and OS were calculated with the Kaplan-Meier method and the Log-Rank test. First, DFS and OS were compared according to age, gender, disease stage, smoking status, history of adjuvant chemotherapy and/or adjuvant radiation therapy, type of surgery, histological subtype, ECOG performance score, and membranous staining intensity by univariate cox regression analysis, and then multivariate analysis was performed. Data were expressed as “mean (standard deviation; SD)”, minimum-maximum, 95% confidence interval (CI) and percent (%) where appropriate. P<0.05 was considered statistically significant.

## RESULTS

### Demographic Characteristics

The age, gender, smoking status, histological subtype, stage of the disease, developing metastasis, and median survival times of the patients are summarized in Table 1.

**Table 1:** Demographic characteristics of the patients

**Group**	**Subgroup**	**Number (%)**
**Age**	Mean±SD	58±8
	Age range	35-76
**Gender**	Male	79 (87.8)
	Female	11 (12.2)
**Smoking status**	Smoker	74 (82)
	Non-smoker	16 (17)
**Histological subtype**	Adenocarcinoma	33 (36)
	Squamous cell carcinoma	48 (53)
	Large cell carcinoma	9 (10)
**Stage**	Stage-1	28 (31)
	Stage-2	41 (45)
	Stage-3	21 (23)
**Metastasis status**	Lymph node metastasis	29 (32)
	Bone metastasis	15 (17)
	Contralateral lung metastasis	13 (14)
	Brain metastasis	13 (14)
	Liver metastasis	9 (10)
	Adrenal metastasis	3 (3)
**Median survival time**	Disease-free survival (months)	25 (17.8-32.2)
	Overall survival (months)	46 (28.6-63.4)
**Final situation**	Still followed	50 (55)
	Died	40 (44)

ECOG performance scale (PS) assessment revealed that 49 (54%) of the patients were PS-0, 39 (43%) were PS-1 and 2.

Overall, 52 (57%) patients had lobectomy, 36 (40%) had pneumonectomy, and 2 patients had segmentectomy. Eighteen patients received radiotherapy and 66 patients received adjuvant chemotherapy.

### Expression of PD-L1 and its Relationship with the Clinicopathological Features

PD-L1 was positive (tumor staining ≥ 5%) in 34 (37.0%) patients. Based on membranous staining intensity, PDL1 was negative in 56 patients, 1+ in 20 patients, 2+ in 11 patients, and 3+ in 3 patients. According to the membranous staining scores 14 (15.0%) patients were considered to be PD-L1 positive.

Based on H-Score analysis, 5 (5%) patients were considered to be positive (H-scores ≥ 50%).

No significant difference was noted between PD-L1 negative and PD-L1 positive patients in terms of age (Mean age 58 ± 8.3 vs. 55 ± 8.5 years, p=0.149).

In all these assessment methods, both DFS and OS were shorter in PD-L1 positive patients. PD-L1 expression frequency and DFS and OS times according to various criteria are summarized in Table 2.

**Table 2:** Prevalence of PD-L1 by various criteria and its effect on survival

**Evaluation Criteria**	**Subgroup**	**Number (%)**	**DFS (95% CI)** **(months)**	**p**	**OS (95% CI)** **(months)**	**p**
**Membranous staining intensity** **(2+ and 3 + positive)**	Positive	14 (15)	20 (4.5-35)	0.340	24 (2.2-68)	0.707
	Negative	76 (85)	27 (20-33.6)		46 (39-52)	
**Tumor staining percentage** **(> 5% positive)**	Positive	34 (37)	20 (4.8-35)	0.437	28 (2-55)	0.547
	Negative	56 (63)	27 (16.6-37)		46 (38-53)	
**By H-Score** **(> 50% positive)**	Positive	5 (5)	20 (1.2-38.7)	0.208	24 (9.9-38)	**0.049**
	Negative	85 (95)	27 (17.5-36)		48 (33.6-62)	

There was no statistically significant association between PD-L1 expression and clinicopathological features such as the patient’s age, gender, smoking status, tumor stage and histological subtype. When the relationship between clinicopathological features and PD-L1 expression was examined, the intensity of membranous staining was taken as basis. These findings are summarized in Table 3.

**Table 3:** Evaluation of clinicopathological features of the patients with PD-L1 expression

**Clinicopathological parameter**	**Subgroup**	**PD-L1 positive, n=14 (%)**		**PD-L1 negative, n=76 (%)**		**p-value**
**Age**	<65	12	85	57	75	0.506
	>65	2	15	19	25	
**Gender**	Male	14	100	65	85	0.202
	Female	-	0	11	15	
**Disease Stage**	Early (Stage 1-2)	10	71	59	77	0.732
	Late (Stage 3)	4	29	17	22	
**Histological type**	Adenocarcinoma	3	22	30	39	0.332
	Squamous cell type	10	71	38	50	
	Large cell cancer	1	7	8	11	
**Smoking status**	Smoker	13	92	61	80	0.450
	Non-smoking	1	8	15	20	

Pearson Chi-Square Test

While patients were operable and had no metastasis at the time of diagnosis; various distant metastases developed in some patients during follow-up, including lymph node metastasis in 29 (32%), bone metastasis in 15 (17%), contralateral lung metastasis in 13 (14%), brain metastasis in 13 (14%), liver metastasis in 9 (10%), pleural metastasis in 7, and adrenal gland metastasis in 3 patients. There was no statistically significant association between PD-L1 expression, and the metastasis status of the patient.

### PD-L1 Expression and Survival Outcomes

**Figure 2:**
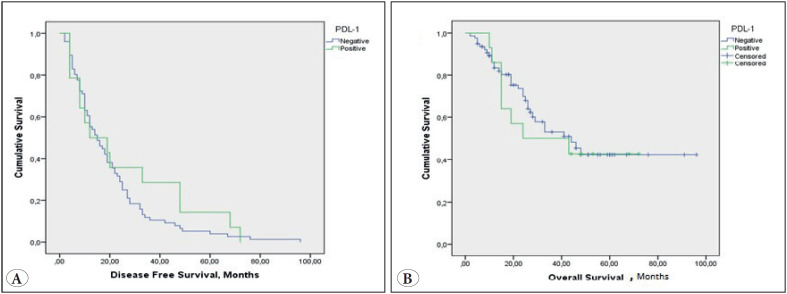
Kaplan Meier plots showing the Disease-Free Survival (DFS) and Overall Survival (OS) times of Programmed death-ligand 1 (PD-L1) positive and negative patients by membranous staining intensity. **A)** DFS curves in PD-L1 positive and negative NSCLC patients. **B)** OS curves in PD-L1 positive and negative NSCLC patients.

Based on the analysis of membrane staining intensity of PD-L1 expression, the median DFS time was 20 months (95% CI 4.54-35.45), 27 months (95% CI 20.35-33.68) and 25 months (95% CI 17.85-32.14) in PD-L1 positive, PD-L1 negative, and total patients, respectively. PD-L1 status had no significant impact on DFS (p=0.340). OS was also similar between PD-L1 positive and PD-L1 negative patients according to membranous staining intensity 24 months (95% CI 2.2-68) vs. 46 months (95% CI 39-52), respectively (p=0.707). Kaplan-Meier plots showing the DFS and OS times of PD-L1 positive and negative patients according to membranous staining intensity are shown in Figure 2.

Based on the analysis of the H-Score, no significant difference was noted in DFS between PD-L1 positive and PD-L1 negative patients (median 20 [95% CI 1.2-38.7] months vs. median 27 [95% CI 17.5-36] months, respectively (p=0.208)), whereas OS time was significantly shorter in PD-L1 positive vs. PD-L1 negative patients (median 24 months [95% CI 9.9-38] vs. 48 months [95% CI 33.6-62.3], (p=0.049)).

Cox regression analysis findings on potential factors that can affect DFS (age, gender, membranous staining intensity of PD-L1, disease stage, smoking status, adjuvant chemotherapy status, adjuvant radiotherapy status, type of surgery, histological subtype, and patient performance) are summarized in Table 4.

**Table 4:** Univariate and multivariate analysis of factors affecting disease-free survival

**Clinicopathological Parameter**	**Univariate analysis**			**Multivariate analysis**		
	**HR**	**CI (95%)**	**p**	**HR**	**CI (95%)**	**p**
Age (≥65 or <65)	1.329	0.628-2.813	0.457			
Gender (male or female)	1.220	0.434-3.434	0.706			
Smoking status (smoker or non-smoker)	1.046	0.462-2.370	1.046			
PD-L1 staining intensity (positive or negative)	1.451	0.667-3.156	0.348			
H-Score for PD-L1 (positive and negative)	1.909	0.678-5.373	0.221			
Stage (advance stage or early stage)	4.506	2.199-9.582	**0.001**	5.22	2.40-11.37	**0.01**
Adjuvant RT (present or not)	2.394	1.167-4.912	**0.017**	1.52	0.63-3.63	0.34
Adjuvant CT (absent or present)	2.292	1.210-4.342	**0.011**	2.19	1.13-4.23	**0.02**
Type of surgery (pneumonectomy or lobectomy)	8.493	1.902-39.91	**0.022**			
Histological subtype (adenocarcinoma or squamous cell cancer)	7.239	0.978-53.59	0.533			
Performance (2 or 1-0)	5.469	1.234-24.23	**0.025**	2..33	1.21-4.50	**0.01**

**RT:** Radiotherapy, **CT:** Chemotherapy, **HR:** Hazard Ratio, **CI:** Confidence Interval Cox-regression Method

When the parameters affecting the OS were examined with univariate analysis, the stage of the disease, the PD-L1 positivity according to the H-score, the adjuvant RT status, the type of surgery performed, and the patient’s performance status were found to be significantly associated with OS. In multivariate analysis, the stage of the disease and the patient’s performance status were found as independent parameters affecting OS (Table 5).

**Table 5:** Univariate and multivariate analysis of factors affecting overall survival

**Clinicopathological Parameter**	**Univariate analysis**			**Multivariate analysis**		
	**HR**	**CI (95%)**	**p**	**HR**	**CI (95%)**	**p**
Age (≥65 or <65)	1.198	0.567-2.534	0.636			
Gender (male or female)	1.497	0.460-4.867	0.503			
Smoking status (smoker or non-smoker)	1.002	0.440-2.284	0.996			
PD-L1 staining intensity (positive or negative)	1.160	0.532-2.529	0.710			
H-Score for PD-L1 (positive and negative)	2.722	0.952-7.782	**0.049**			
Stage (advance stage or early stage)	3.278	1.620-6.630	**0.001**	2.97	1.45-6.09	**0.01**
Adjuvant RT (present or not)	2.060	1.010-4.198	**0.047**			
Adjuvant CT (absent or present)	1.572	0.829-2.981	0.166			
Type of surgery (pneumonectomy or lobectomy)	6.421	2.56-49.46	**0.013**			
Histological subtype (adenocarcinoma or squamous cell cancer)	5.484	0.716-42.07	0.101			
Performance (2 or 1-0)	2.234	1.688-34.13	**0.008**	1.98	1.02-3.85	**0.01**

**RT:** Radiotherapy, **CT:** Chemotherapy, **HR:** Hazard Ratio, **CI:** Confidence Interval Cox-regression Method

## DISCUSSION

In this study, we investigated the prevalence of PD-L1 expression in NSCLC patients and its relationship with clinicopathological features along with its predictive and prognostic value.

In previous studies, cases with a tumor staining percentage of ≥5% or membranous staining intensity of 2+ and 3+, or an H-Score of ≥50% were considered positive for PD-L1 (12). In our study, the prevalence of PD-L1 in NSCLC patients was determined to be 37%, 15% and 5% based on tumor staining percentage, membranous staining intensity and H-score, respectively. The H-score from all patient samples was used as the cut-off value to standardize PD-L1 positivity. Given the use of various threshold limits for PD-L1 positivity, its prevalence has varied among different studies (13,14), ranging from 5-57%, depending on the method, scoring system, or antibody used, as well as the differences in race, histological subtype and disease stage (15). The significant efficacy of atezolizumab developed for PD-L1 targeting therapy was demonstrated in the group of NSCLC patients with high PD-L1 expression (16). Therefore, the presence of PD-L1 expression in the NSCLC patient group is an important marker in predicting treatment efficacy in PD-L1 targeting therapy.

Although there are many studies on PD-L1 expression in NSCLC patients, its relation to clinicopathological features remains unclear. In our study, no statistically significant relationship was found between PD-L1 expression and patients’ age, gender, or smoking status. However, PD-L1 expression was relatively higher among men, patients that are <65 years, patients with early stage disease, and squamous cell cancer. In previous studies, in general, PD-L1 protein expression was not significantly associated with clinicopathological features of NSCLC, including gender, age, and smoking status (17). However, in one study (18), PD-L1 expression was reported to be associated with smoking status in NSCLC patients. In another study, PD-L1 expression was found to be high in non-smokers and women in the Japanese population (19). This difference may be due to the fact that the rate of women and non-smokers in the study conducted in the Japanese population was significantly higher than the rates in our study. In a study by Cooper et al., PD-L1 expression was found to be high in the young patient group (14). In this case, it is thought that tumor tissue in young patients expresses PD-L1 in response to the strong immune system. The relationship between PD-L1 and clinicopathological features in NSCLC patients is not as clear as the relationship between driver mutations and clinicopathological features.

Our findings related to the membrane staining intensity revealed 14 patients to have positive PD-L1 expression, including 10 (71%), 3 (22%), and 1 (7%) patient with squamous cell carcinoma, adenocarcinoma and large cell carcinoma, respectively. In accordance with the majority of the previous studies, there was no statistically significant association between PD-L1 expression and histological subtypes in our study. However, the positivity of PD-L1 expression was relatively higher in squamous cell carcinoma according to the staining density. PD-L1 expression was reported to be higher in adenocarcinoma in a study by Mu et al. (20), whereas it was reported to be associated with squamous cell carcinoma in a study by Velcheti et al. (21). However, in a meta-analysis of 1550 patients by Pan et al., the authors reported no relationship between PD-L1 expression and histological subtypes (17). This may be due to the fact that studies are conducted with patient groups with different stages and different clinicopathological characteristics. Squamous cell lung cancer benefited less from drugs developed against driver mutations. Therefore, high PD-L1 expression and benefit from immunotherapy are important in this subgroup of patients.

The relationship between PD-L1 expression and the stage of the disease in some cancers has been previously studied (22). In our study, there was no significant relationship between PD-L1 expression and disease stage when stage-1 and 2 were considered as early stage and stage-3 as local-advanced stage. However, numerically higher PD-L1 expression was observed in early stage patients. Perhaps in the early stage of the disease, PD-L1 expression of tumor cells increases as a response to the strong immune system. In previous studies, no significant relationship was reported between PD-L1 expression and disease stage (20). The findings of the current study were also compatible with the literature. In addition, the high PD-L1 expression in the early stage in our study may be related to the shorter DFS time in the PD-L1 positive group.

There is a hypothesis that when PD-L1 is positive, the tumor can hide itself from the immune system and spread in a shorter time and the disease may become metastatic (23). Therefore, in our study, PD-L1 expression and distant metastasis status were investigated to determine whether the high PD-L1 expression is related to the recurrence of the disease or the increased risk of distant metastasis. Although our patients were operable at the time of study enrollment, various cases developed metastasis during the follow-up. The distant organ metastasis involved bone, brain and liver metastasis, supporting the literature (24). Our findings revealed no significant association between the metastasis status of the patients and PD-L1 expression. The high expression of PD-L1 suggests an increase in the spread of the disease. In a past study, a higher rate of lymph node metastasis was found in the patient group with high PD-L1 expression (25). In addition, a high expression of PD-L1 was considered to indicate the aggressiveness of the tumor and to be an independent risk factor for postoperative recurrence (26). When NSCLC is PD-L1 positive, it is not clear which organ metastases increase significantly. This was difficult to detect in our study population.

In previous studies, the effect of PD-L1 expression on DFS and OS in NSCLC patients was investigated (27). In most studies, increased PD-L1 expression was found to be associated with a short life span. On the other hand, it was not significantly associated with survival in some studies and reported to be a good prognostic factor in others (23). In our study, according to the membrane staining intensity, the median DFS was 20 months (95% CI 4.5-35.4) in PD-L1 positive patients, while it was 27 months (95% CI20.3-33.6) in PD-L1 negative patients, with no statistically significant difference between the two groups. OS was 24 months (95% CI 2.4-68) in patients with positive PD-L1 expression and 46 months (95% CI 39-52) in patients with negative PD-L1 expression, and no statistically significant difference was noted in OS time between the two groups. Some parameters did not reach statistical significance in our study due to small sample size and insufficient follow-up time. However, when evaluated according to the H-Score, OS time was significantly shorter in PD-L1 positive vs. PD-L1 negative patients (24 months [95% CI 9.9-38] vs. 48 months [95% CI 33.6-62.3], p=0.049). DFS time was also shorter in the PD-L1 positive vs. negative group, although it was not statistically significant.

OS time was significantly shorter in the PD-L1 positive group according to the percentage of staining, staining intensity, and H-score. In this case, the positive PD-L1 in operated NSCLC patients may require a closer follow-up regarding recurrence. Therefore, in our study, PD-L1 positivity was accepted as a poor prognostic factor for NSCLC patients, similar to previous studies (7). Atezolizumab, avelumab and durvalumab were developed for treatment against this marker, which normally has poor prognostic value, and this was turned into an opportunity. The predictive value of PD-L1 expression has been used in drug efficacy studies of anti-PD-L1 drugs (28).

In the univariate analysis, the factors significantly affecting DFS were the stage of the disease, adjuvant chemotherapy, adjuvant RT, the performance status of the patients, and the type of surgery performed. In the multivariate analysis, the stage of the disease, adjuvant chemotherapy, and the performance status of the patients were significant predictors of DFS. In univariate analysis, factors affecting OS were stage of disease, PD-L1 positivity according to H-score, adjuvant radiotherapy, type of surgery, and patient performance. The factors affecting OS in the multivariable analysis were the stage of the disease and the patient’s performance status. These findings of our study were consistent with previous studies (29–31).

Limitations associated with this study were related to its retrospective design and relatively small sample size. In addition, there was a possibility of cross-reactivity since polyclonal antibodies were used in our study. However, the results of our study should contribute to new prospective studies on the predictive and prognostic role of PD-L1 expression in patients with NSCLC, as assessed by immunohistochemistry. It will be more appropriate to conduct studies with standard methods and antibodies in large cohorts and homogeneous patient groups.

In conclusion, our findings revealed that the prevalence of PD-L1 expression in NSCLC patients changes depending on the evaluation methods and there is no well-established relationship between PD-L1 expression and clinicopathological features. When PD-L1 expression is evaluated using standard methods, it may have predictive value for anti-PD-L1 treatment modalities. PD-L1 positivity is associated with shorter DFS and OS in operated NSCLC patients. PD-L1 expression in operated NSCLC patients may require closer follow-up due to the risk of early recurrence. Accordingly, our findings seem to indicate PD-L1 expression as a poor prognostic marker for NSCLC.

## Conflict of INTEREST

The authors do not have any conflict of interest or financial disclosures to declare.
